# Do Embedded and Peripheral Corporate Social Responsibility Activities Lower Employees’ Turnover Intentions?

**DOI:** 10.3389/fpsyg.2022.926962

**Published:** 2022-06-24

**Authors:** Yumin Liu, Kamran Ijlal, Muhammad Shehzad Hanif, Aitzaz Khurshid, Zeeshan Ahmed

**Affiliations:** ^1^School of Business, Zhengzhou University, Zhengzhou, China; ^2^UCP Business School, University of Central Punjab, Lahore, Pakistan

**Keywords:** embedded CSR, peripheral CSR, stakeholder theory, SEM, employee turnover

## Abstract

Corporate social responsibility (CSR) remains a topic of interest for both theory and practice due to its multifaceted avenues and potential for growth. We have chosen embedded CSR and peripheral CSR measures to evaluate how these activities affect the employee turnover intentions *via* a mediation mechanism of organizational citizenship behavior (OCB). In doing so, this study addresses important stakeholder concerns and provides meaningful managerial contributions for the employers to encourage more employee participation (through lowering turnover intention) toward sustainable corporate performance. This study incorporates four hypotheses that are tested in a structural equation modeling framework by employing Warp-PLS software. Data were collected from 297 employees working in firms that are renowned for their CSR initiatives. We found support for our key hypotheses leading to strong theoretical contributions to the stakeholder theory. We have addressed the main issues of stakeholders’ response to the CSR tradeoffs and have tried to develop a deeper understanding of managers in initiating peripheral and embedded CSR activities for their firms.

## Introduction

Corporate social responsibility (CSR) persists to be an area of interest for the industry as well as for academia. One of the recent and innovative conceptualizations of CSR (Fu et al., 2022) is in the form of embedded and peripheral CSRs. In today’s corporate environment, firms have multiple stakeholders with varying significance. It is a constant struggle for businesses to recognize a balanced representation of diverse stakeholder groups’ interests and expectations in their CSR policies (Carroll, 1979; Freeman, 2010; Oates and Kloot, 2014). Firms need to motivate their employees to innovatively tackle sustainability challenges by working on the three dimensions of sustainability and handling the interests of diverse stakeholders (Hanif et al., 2018; Abbas et al., 2019a). The term stakeholder became well-known in the management literature because of Freeman’s (1984) work, which sparked a heated debate concerning stakeholders’ roles, relevance, and legitimacy (Mitchell et al., 1997). A recent debate in the stakeholder theory argues for more behavioral orientation instead of the economic value alone (Freeman et al., 2020). In the sustainability literature, various stakeholders lie at the core of the three dimensions of sustainability. The social dimension relates to the employees and suppliers of the firm, the economic dimension relates to the shareholders of the firm, and environmental sustainability relates to the society at large.

Two of the most prominent stakeholders include the community and the employees of the firm (Turker, 2009). Firms perform various CSR activities including peripheral and embedded CSR activities to engage their stakeholders. It is interesting to figure out which type of CSR matters more for employees. Many firms perform peripheral CSR activities to earn a good image, but is it enough to keep your employees engaged? Employee engagement is fundamental to achieve pro-environmental behavior, and hence, this study evaluates how peripheral and embedded CSRs affect the turnover intentions of the employees through the mediating role of organizational citizenship behavior (OCB).

The following research questions are addressed in this study:

Which CSR influences the turnover intention of employees more; is it the peripheral CSR or the embedded CSR?

Is there a mediated relationship between CSR and turnover intentions of the employees through OCB?

The remainder of this research is organized as follows. The ideas from the CSR, OCB, and stakeholder theory are presented in the section “Introduction.” In the section “Literature Review,” these ideas are combined to construct appropriate hypotheses regarding how employees view the embedded and peripheral CSR measures and how they affect their turnover intentions through the mediating role of OCB? The methodology is presented under “methodology” section followed by the analysis of measurement & structural model. Results are discussed in the “Discussion” section with the conclusion and research limitations coming in the end in “conclusion” section.

## Literature Review

### Stakeholder Theory

The stakeholders of a firm are defined as “the individuals and constituencies that contribute, either voluntarily or involuntarily, to (the firm’s) wealth-creating capacity and activities, and that are therefore its potential beneficiaries and/or risk bearers” (Post et al., 2002). The stakeholder theory proposes that stakeholder relationships are at the core of managerial decision-making (Freeman, 1984).

Although many social players are stakeholders of a firm, scholars have tried to address why certain stakeholders are more important to the firm as compared to others (Muzaffar et al., 2019). In this regard, many classifications are presented in the literature. Clarkson (1995) has suggested a distinction between the primary and secondary stakeholders based on their influence on the organization. Shareholders, customers, employees, and suppliers represent the primary shareholders of the firm (Khurshid and Ahmed, 2020). Another classification of the voluntary and involuntary stakeholders is suggested by Mitchell et al. (1997). Voluntary stakeholders have invested a substantial amount of money and resources in the firm.

Employee attitudes of a firm toward its CSR activities are influenced by the way it treats its stakeholders (El Akremi et al., 2018). The community of the firm and its employees are the firm’s two most important stakeholders. When the firm performs the embedded CSR activities, it performs its CSR activities on its employees, and when it performs the peripheral CSR activities, it performs its CSR activities on the society.

### Embedded and Peripheral Corporate Social Responsibility

The literature on CSR has expanded exponentially in size and complexity due to its current applicability. In curtailing the prevailing crises caused due to COVID-19, novel activities beyond the non-pharmaceutical intervention were strongly recommended (Zhou et al., 2021), suggesting that innovative CSR activities also hold a lot of promise to play their part. Apart from the social aspect, CSR holds important financial implications for the firms (Ang et al., 2022). Several reviews of CSR are available (Carroll and Shabana, 2010; Peloza and Shang, 2011; Frynas and Yamahaki, 2016) and a parallel universe seems to exist due to a variety of conceptualizations of CSR (Aguinis and Glavas, 2012). Researchers in various disciplines have conceptualized CSR through different frameworks and levels of analysis, and many studies link CSR with sustainability (Kolling et al., 2022), while a growing body claims that the social dimension of sustainability remains understudied (Desiderio et al., 2021). In this study, a relatively newer and innovative conceptualization of CSR is taken into account of embedded CSR and peripheral CSR proposed by Aguinis and Glavas (2012). This conceptualization offers an innovative lens and analysis of the previous literature (Liu et al., 2021; Ge et al., 2022). This categorization applies to both the antecedents and outcomes of CSR, overcoming the limitations of the previous ones. The present classification is based on Aguinis’s (2011) definition of CSR, which understands CSR activities as “context-specific organizational actions and policies that take into account stakeholders’ expectations and the triple bottom line effect of economic, social, and environmental performance.”

Embedded CSR is based upon the core competencies of a firm and attempts to integrate CSR within the firm’s operations, routines, and strategy. In contrast to embedded CSR, peripheral CSR does not integrate CSR within the firm’s operations, routines, and strategy. A simple conceptualization of embedded CSR is to focus within the organization and of peripheral CSR is to focus externally. In the present study, embedded CSR is studied as CSR activities performed on the employees of the firm, and peripheral CSR as CSR activities performed on the society.

Stakeholders respond to the firm’s responsible behaviors toward other stakeholder groups as well as their group (Rupp et al., 2013). The literature also illustrates that businesses must frequently tradeoff the interests of many stakeholders (Reynolds et al., 2006; Rupp et al., 2006). However, it is unknown how these stakeholders would react to the tradeoffs between self-directed and other-directed CSRs, in this case, the embedded CSR activities and the peripheral CSR activities. The outcomes of a few empirical research comparing self- vs. other-directed CSR are varied (Peloza and Shang, 2011).

These varied findings imply that personal material gains of the stakeholders from other-directed CSR are not the only source of value for them. As a result, we expand on the existing knowledge to better explain the motivations and mechanisms that act as a driving force to employees’ tradeoffs between embedded and peripheral CSRs by hosting the element of OCB.

### Organizational Citizenship Behavior

Organizational citizenship behavior is defined as “innovative and spontaneous behaviors” for the organizational success that is not included in the formal job description (Katz, 1964), whereas Organ (1988) states it as an “individual behavior that is discretionary, not directly or explicitly recognized by the formal reward system, and that in the aggregate promotes the effective functioning of the organization.” OCB has emerged as an interesting area of research, where many scholars have attempted to gauge the impact of OCB and servant leadership (Asad et al., 2017; Asada et al., 2020) on various organizational performance dimensions (Mubeen et al., 2021). What makes it intriguing is to find the factors that lead to this important behavior, irrespective of the formal reward system.

Organizational citizenship behavior has been studied as an antecedent of firm performance (Skarlicki and Latham, 1995; Lowery and Krilowicz, 1996; Podsakoff et al., 1997) and sustainability (Qing and Jin, 2022). It is argued that sustainability is not possible without the element of OCB within the employees of the firm (Wang et al., 2017). Similarly, the antecedents of OCB have also been explored. Some of the antecedents of OCB include personality (Organ and Konovsky, 1989), perceived fairness (Moorman, 1991), and servant leadership (Smith et al., 1983) analyzed through the lens of social exchange theory. There is still room to explore more relevant antecedents of OCB and we validate the argument with the testing of whether embedded CSR or peripheral CSR leads to turnover intentions through OCB or not?

### Turnover Intention

Turnover intention is “an individual’s behavioral intention or conation to leave the employment of the organization” (Fishbein and Ajzen, 1977). Turnover intention is defined by Lacity et al. (2009) as “the extent to which an employee plans to leave the organization.” Another definition by Tett and Meyer (1993) is “the conscious and deliberate willfulness to leave the organization.” In all of the definitions cited, we see the intentions, willfulness, and plans of the employee to leave the organization in which he or she is presently employed.

How CSR perceptions influence turnover intentions of employees has been studied earlier with mixed results. Some studies found no relationship between the two (De Gilder et al., 2005), and these discrepancies suggest that CSR perceptions may have an indirect influence on turnover intentions. Trust (Hansen et al., 2011), organizational commitment (Hollingworth and Valentine, 2014), and organizational identification (Jones, 2010) are among the mediators that have been studied in the past, and in recent studies, OCB has also been studied as a direct antecedent of employee turnover intentions (Manoppo, 2020; Li and Xie, 2021). To open the black box, we have studied CSR in two different constructs and engaged the OCB as a mediator to examine turnover intentions.

## Theoretical Framework

A review of the extant literature advocates that CSR can help a firm not only engage new stakeholders but also deepen the ties with its current stakeholders (Turban and Greening, 1997; Barnett, 2007). Employees, customers, suppliers, and investors are primary stakeholders who provide vital resources to the firm and associate willingly with it, implying that attracting and retaining these stakeholders, especially the employees, is critical for corporate performance (Zhang et al., 2012).

The general trend of organizational CSR initiatives results in favorable employee-oriented organizational outcomes (Burbano, 2016; Flammer and Luo, 2017; Yan et al., 2021). Carnahan et al. (2017) has discussed two main reasons for the CSR activities that result in higher retention of employees. First, participating in peripheral CSR initiatives can help a company develop a prosocial reputation (De Roeck and Delobbe, 2012). This reputation could help the company find more candidates (Greening and Turban, 2000). Larger pools of candidates facilitate the firms to source candidates that have a good “fit” with the organization (Kristof−Brown et al., 2005) and therefore have a lower tendency to depart. Furthermore, association with an organization that is being regarded as a “good” performer by outsiders may bring positive utility to the employees (Brammer et al., 2007; Badar and Irfan, 2018), bringing the chances of them quitting to minimal after controlling other influences. This discussion forms the basis of the hypothesis connecting peripheral CSR activities with the turnover intentions of the employees.

Corporate social responsibility efforts may also minimize employee turnover by boosting the meanings individuals derive from their work. The embedded CSR activities are expected to influence the meaningfulness by increasing the meaningfulness “in” and “at” the workplace. Employees may experience a feeling of meaning “in” their work by participating in the CSR activities such as volunteer programs, which allow them to make a direct and visible social impact. Employees may have a greater sense of purpose in those firms that engage in more embedded CSR activities (Azizi et al., 2021), partly because the embedded CSR initiatives mirror a more favorable prosocial environment and culture. Employees’ identification with and dedication to the company may improve as a result of this greater feeling of meaning at work, resulting in a fewer turnover.

**FIGURE 1:**
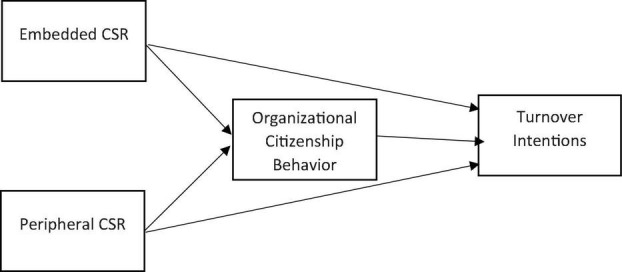
Proposed model.

As a result, a growing body of organizational research postulates a positive influence of CSR investments in the forms of peripheral and embedded CSR activities of an organization upon employee retention. It will be more meaningful for organizations to know whether embedded or peripheral CSR is more influential in reducing employee turnover. On the basis of the proposed framework of this study (see Figure 1); the following hypotheses are presented in light of the foregoing debate:

Hypothesis 1: Embedded CSR initiatives of the firm have a negative impact on its employee turnover intentions.
Hypothesis 2: Peripheral CSR initiatives of the firm have a negative impact on its employee turnover intentions.

There is support for the earlier research of OCB as a consequence of CSR activities (Zhang et al., 2014). Prior studies that have used explicit avoidance behaviors, including tardiness and absenteeism, are not considered as appropriate predictors of turnover intentions because the employees would avoid such explicit behaviors that would jeopardize their compensation and related benefits (Chen et al., 1998; Afzal et al., 2020). Chen et al. (1998) further suggest that a discretionary behavior like OCB is a better predictor of turnover intentions as compared to explicit behaviors. Podsakoff et al. (1997) support this argument that OCB may lead organizations to retain their employees. Furthermore, He et al. (2019) found OCB to mediate the relationship between CSR activities and task performance.

Apart from sourcing explicit support for the hypotheses, stakeholders’ responses to CSR tradeoffs between self-directed and other-directed CSR remains a relatively unexplored area with mixed results (Peloza and Shang, 2011). In our case, embedded CSR activities are the self-directed CSR activities of the firm, and peripheral CSR activities are the other-directed CSR activities for the employees of the firm. The multi-motive framework by Rupp et al. (2011) have gauged the reaction of employees to the other-directed CSR activities in the presence of uncertainty reduction, moral motives, and relational motives. It is quite relevant to demystify the behavior of the employees by adding the OCB as a mediator between the CSR initiatives and employees’ turnover intentions. The nature of mediation will also shed some light on employees’ turnover intentions concerning their OCB when the firm performs embedded CSR activities as compared to peripheral CSR activities. Combining prior empirical research gaps, we can infer a mediating role of OCB between CSR and turnover intentions. These arguments lead to the following hypotheses:

Hypothesis 3: Embedded CSR negatively influences the turnover intentions of the employees through the mediating role of OCB.
Hypothesis 4: Peripheral CSR negatively influences the turnover intentions of the employees through the mediating role of OCB.

## Methodology

### Sample Size

We utilized purposive sampling for the present study as the population that we intended to cover was unknown. According to Soper (2020) sample size recommendations, the minimum sample size calculations for the present study were found to be 241 based on the number of latent constructs being 4 with the number of observed items being 25, while the probability level was 0.05, with desired statistical power at the conventional 0.80 level with an anticipated effect size of a medium level, i.e., 0.3.

### Choice of a Representative Sample

Since our research is focused on the various dimensions of CSR, we decided to focus on the major players from different industry sectors who led various CSR initiatives in the local society. Based on the proceedings of the 11th Corporate Social Responsibility Summit held in the Serena Hotel, Islamabad, Pakistan on January 24, 2019, 55 different organizations were awarded for their active participation in championing the CSR practices; we decided to approach the top 12 different organizations coming from various industry sectors like information and communication technology, home appliances manufacturing, textile, leather, food and beverages, etc.

It is pertinent to note that these organizations were all leading local manufacturers that showed an active involvement in responsible manufacturing, production, and operations along with participation in the community development for social and societal benefits. For example, one firm that participated in our study is active in welfare activities for flood relief and earthquake emergencies. It also arranges large-scale voluntary medical services and relief camps for the deprived communities. The company has demonstrated an active role in the community through embedded CSR measures by establishing an HSE department in their office. Some organizations are involved in CSR initiatives such as providing food and shelter for underprivileged children, providing aid for disaster management, and providing aid in rehabilitation of victims. Yet, there are also some other organizations participating in fighting pandemics, like Dengue and Swine flu; some firms have taken initiatives to control the greenhouse effect. They have adopted environmental management accounting measures through green packaging, recycling, and other allied steps to safeguard the environment.

We approached the concerned member designated in the firm office to help us with the data collection. The questionnaires were distributed to the relevant members of various organizations and we executed two drives for the data collection. The campaigns at different times helped us control for different biases. The data collection was done through physical as well as electronic surveys.

We delivered a total of 700 questionnaires to various employees from these organizations, out of which we received back 297 completed questionnaires comprising 114 online responses, while the remaining 183 were hard copies collected from different offices. Upon scrutiny of these received forms, 20 responses were discarded due to either incomplete or manipulated responses while 9 more were not considered due to unengaged responses making it a tally of 269 cases for the final analysis in Warp PLS-7. The demographic characteristics of our respondents are provided in Table 1.

**TABLE 1:** Respondent profile.

Demographic variables			

Variable	Category	No of responses	% age
Gender	Men	201	74.7
	Women	68	25.3
Age	18–25	31	11.5
	26–30	107	39.8
	31–35	86	32
	36–40	27	10
	>40	18	6.7
Qualifications	Undergraduate	18	6.7
	Graduate	72	26.8
	Masters	179	66.5
Work experience	<6 months	6	2.2
	6 months to 1 year	14	5.2
	1–2 years	32	11.9
	3–5 years	62	23
	>5 years	155	57.6
*N* = 269			

### Questionnaire Design

The instrument design for our survey was based on the validated measures from the past literature. The details of the constructs are discussed separately below.

#### Peripheral Corporate Social Responsibility

Peripheral CSR was measured using the scale developed by Turker (2009). Six items measured social and non-social CSR (CSR to community-based on a 5-point Likert scale with anchors ranging from strongly agree to strongly disagree).

#### Embedded Corporate Social Responsibility

Embedded CSR was measured based on the scale introduced by Turker (2009). Six items focused on CSR to employees with anchors ranging from strongly agree to strongly disagree.

#### Organizational Citizenship Behavior

The OCB was measured using the Kumar and Shah’s (2015) scale, which is adapted from the Podsakoff et al.’s (1997) instrument. Data were collected from the participants based on a 5-point Likert scale with 1 = Strongly Disagree and 5 = Strongly Agree.

#### Turnover Intention

Bothma and Roodt (2013) developed the TIS-6 based on Osgood’s (1964) semantic differential technique of using a series of bipolar 5-step response scales defined by the two opposites (e.g., never–always; to no extent–to a very large extent; highly unlikely–highly likely). A sample item of the 5-item scale is “How often have you considered leaving your job?” For this study, the responses of the participants were taken on a 5-point scale ranging from 5 = highly likely to 1 = highly unlikely.

### Assessment of Measurement Model

All the constructs used in this study were adapted from the literature; however, we made minor changes to fit them according to our research. The measurement model was assessed for the validity measurements, i.e., the discriminant and convergent validity measures were inspected. All the discriminant validity measures, i.e., the square root values of AVE for each construct are provided in Table 2. The presence of multicollinearity was also observed to be below the threshold value of 3.3, according to the recommendation in the literature (Kock, 2015).

**TABLE 2:** Discriminant validity results for different constructs.

	CSR-Prpl	CSR-Embd	OCB	TO-INT
CSR-Prpl	(0.768)			
CSR-Embd	0.709	(0.781)		
OCB	0.421	0.476	(0.683)	
TO-INT	−0.417	−0.487	−0.731	(0.84)

*Square roots of average variances extracted (AVEs) are shown in the bracketed text on the diagonal.*

The variance-based SEM techniques should be carefully chosen based on the particular conditions of the available data. These criteria normally involve the assessment of sample size, data normality, etc. (Hanif et al., 2021). This research intended to test the direct effect of both embedded CSR and peripheral CSR on the turnover intentions of the employees along with the mediating relationships through OCB. We ran checks for data normality and observed some non-normal data. Hence, we decided to employ Warp-PLS-7 for the analysis, which is a recommended tool in the literature (Kock and Hadaya, 2018). The initial analysis showed that all factor loading for our items ranged from 0.536 to 0.878, meeting the minimum threshold of 0.50, so we moved on with the analysis of the measurement model (Hair, 2009).

We confirm that the Cronbach’s alpha measure for all the constructs met the threshold level α = 0.7, as provided in Table 3. The measured AVE value for the OCB construct is <0.50; however, it is still acceptable because the Cronbach’s α value is well above the threshold value of 0.70 (Fornell and Larcker, 1981).

**TABLE 3:** Reliability and convergent validity.

Construct	Item	Factor loadings	Composite reliability	Value of Cronbach’s α	Average variance extracted	Dijkstra’s PLSc reliability
Peripheral CSR	CSR-P1	0.784	0.896	0.86	0.591	0.872
	CSR-P2	0.794				
	CSR-P3	0.849				
	CSR-P4	0.78				
	CSR-P5	0.689				
	CSR-P6	0.702				
Embedded CSR	CSR-E1	0.771	0.903	0.871	0.61	0.887
	CSR-E2	0.792				
	CSR-E3	0.82				
	CSR-E4	0.782				
	CSR-E5	0.823				
	CSR-E6	0.689				
OCB	OCB1	0.75	0.837	0.766	0.466	0.82
	OCB2	0.797				
	OCB3	0.769				
	OCB4	0.62				
	OCB5	0.579				
	OCB6	0.536				
Turn over intention	TI1	0.75	0.905	0.86	0.706	0.873
	TI2	0.797				
	TI3	0.769				
	TI4	0.62				

Since the common method bias is a general problem associated with the single instrument of measurement, i.e., the survey/questionnaire, different measures were adopted to guard against it. We designed the instrument in such a way that no ambiguities or unfamiliar terms were used and the presentation of questions were made random to avoid unengaged response from respondents as also recommended and used in the previous literature (Podsakoff et al., 2003; Kong et al., 2022). Similarly, the variance inflation factor measurement was assessed to check for the possibility of common method variance. Extant literature recommends that the highest VIF value obtained from the analysis should be below the threshold value of 3.3 to ensure that the analysis is not affected by the common method variance issues (Kock, 2015; Hanif et al., 2021). Hence, all the VIF values were checked and the highest VIF value obtained was observed to be 2.273.

### Analysis of the Structural Model

**FIGURE 2:**
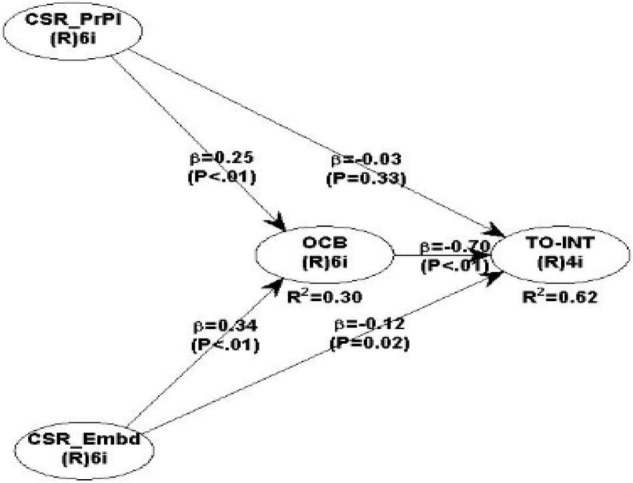
Research framework with path coefficients and corresponding *p*-values.

The results of the final structural model revealed the important values of β-levels and *p*-values for various constructs employed in our model, as shown in Figure 2. The total variance explained using the direct and indirect influence of both peripheral and embedded CSRs was found to be *R*^2^ = 0.62, which is fairly high. The relative path sizes for different constructs are also produced in Table 4. Out of the four hypotheses proposed, this model found support for three of them. The details of the results are produced in Table 4.

**TABLE 4:** Model fitness indices.

Model fit results		

Index	Value	Threshold value
Average block variance inflation factor	1.798	Should be ≤ 3.3
Average full collinearity variance inflation factor	2.202	Should be ≤ 3.3
Avg. path coefficient	0.289	*p*-value should be <0.001
Avg. adj. R-squared AARS	0.460	*p*-value should be <0.001
Avg. adj. R-squared AARS	0.456	*p*-value should be <0.001
Tenenhaus goodness of fit	0.522	Should be large ≥ 0.36
Statistical suppression	1.000	Should be ≥ 0.7
Causality direction ratio (Non-linear bivariate)	1.000	Should be ≥ 0.7
Contribution ratio	1.000	Should be = 1
Simpson’s paradox	1.000	Should be = 1

The model fitness indices are produced in Table 4. All indices met the various goodness of fit criteria for different measures of APC, *R*-squared ARS, AVIF, etc. (Chitsaz et al., 2017; Rasoolimanesh et al., 2017; Hanif et al., 2021).

The results, displayed in Table 5, revealed that the direct path from peripheral CSR to turnover intentions was not significant as the *p*-value is >0.5, leading us to reject hypothesis H1. At the same time, it was observed that the indirect effect of peripheral CSR on the turnover intention through the OCB was significant, thus indicating a full-mediation mechanism through OCB, which supports our hypothesis H3.

**TABLE 5:** Summary of direct, indirect, and total effects.

Identifier	Path	Path coefficient	Standard error	*p*-value	Effect size	Status of hypothesis
**Direct effects**						
A	Peripheral CSR = > TO-Intention	−0.03	0.061	0.33	0.01	Not significant
B	Embedded CSR = > TO-Intention	−0.12	0.06	0.02	0.06	Significant
**Indirect effects**						
A	Peripheral CSR = > OCB = > TO-Intention	−0.177	0.042	<0.001	0.083	Significant
B	Embedded CSR = > OCB = > TO-Intention	−0.24	0.041	<0.001	0.126	Significant
**Total effects**						
	A + a	−0.204	0.059	<0.001	0.09	Significant
	B + b	−0.365	0.057	<0.001	0.191	Significant

Similarly, we found support for H2 where results indicate a significantly strong direct relationship between the embedded CSR and the turnover intentions (*p*-value < 0.05 and effect size >0.02). Additionally, a strong indirect effect was also noted lending the support that OCB is partially mediating the relationship between the turnover intention and embedded CSR (*p*-value being < 0.001 and effect size >0.02).

## Discussion

This study attempt to address the fundamental question of when and how the contemporary CSR conceptualization directs its stakeholders’ sustainable behavior. We have used two measures of CSR, embedded CSR and peripheral CSR, as the antecedents of the OCB and turnover intentions. The results indicate a direct as well as a mediating relationship between embedded CSR and the turnover intentions, whereas in the case of peripheral CSR, we observed an insignificant relationship between peripheral CSR and the turnover intention and a full-mediated relationship between peripheral CSR and the turnover intention through OCB. Prior research have studied how employee satisfaction is influenced by CSR (Brammer et al., 2007; Turker, 2009); however, relatively how important the embedded CSR is as compared to the peripheral CSR for employee’s turnover remains undiscovered, and our research adds to the limited understanding of stakeholders’ responses to the tradeoffs between the self-directed and other-directed CSR.

We address a theoretical concern of finding common grounds in stakeholders with competing interests. At the basic level, turnover intentions of the employees are higher in the case of embedded CSR activities as compared to peripheral CSR activities, which supports the idea that looking at CSR tradeoffs is important. Embedded and peripheral CSRs reflect the interests of different and at times competing stakeholders. We are estimating whether these competing interests lead to the common grounds for promoting OCB and turnover intentions of the employees that lie at the heart of making organizations more innovative and sustainable.

It is, however, interesting to note that stakeholders that are less egocentric will be able to live with both self-directed as well as other-directed CSRs (Bridoux et al., 2016). In our case, employees that develop OCB and therefore are less egocentric show a fully mediated relationship between peripheral CSR and the turnover intentions through OCB reflecting a tolerance for another directed CSR (Abbas et al., 2019b). It primarily means that this other-directed CSR tolerance, in our case the peripheral CSR tolerance, is not possible without OCB.

Our findings imply that OCB is a major mechanism through which tradeoffs influence employees’ reactions: OCB may contribute to the company to help counteract the negative impact of fewer material benefits for employees, particularly for those who are more other-oriented. The managers of the companies that spend on the peripheral CSR activities could use this knowledge to promote OCB within their firms and to communicate with the employees who have a higher OCB to improve the retention of these employees within the firm.

These discoveries reveal important implications for theory and practice. In terms of broader theoretical contributions, this study attempts to address some of the “tensions” identified by Freeman et al. (2020) in their seminal work. The first concern addressed by this study is related to the question of whether the primary goal of the stakeholder theory is to create value for all the parties involved or only for the company. Our research aims to address this point by simultaneously studying the value creation for the stakeholders and the firm. The embedded and peripheral CSR activities create value for the stakeholders, whereas, lower turnover intentions create value for the firm and prepare the grounds for sustainable growth.

Similarly, this study addresses another concern of whether the stakeholder approach promotes primarily cost minimization or opportunity maximization? In this study, we attempted to figure out if the CSR activities that maximize opportunities also lead to cost minimization by lowering the turnover costs.

This study also holds strong managerial implications. In our case, we performed CSR activities on the employees under embedded CSR. We have limited knowledge of how CSR affects the employees, who are the primary stakeholders of the firm. The employees drive the organizations to tackle important agendas like sustainability and also are driven by them through innovative practices. Retaining employees for long periods have an important consideration for all types of firms. An important finding of this study is that the embedded CSR will retain employees for longer periods and that it is partially performed through the OCB that results due to the CSR activities. Retaining employees through OCB primarily means that the firm will be able to filter and retain employees that can take the extra mile to undertake challenges like sustainable growth and open innovation (Abbas et al., 2020).

Peripheral CSR in this research is studied as CSR activities performed in the community. Many firms use peripheral CSR for marketing activities only and are directed toward the community, which is another important stakeholder of the firm. In our study, we found out that the peripheral CSR does not have a direct influence on the turnover intentions. The peripheral CSR works only through OCB, the relationship between the peripheral CSR and the turnover intentions is completely mediated through OCB. This primarily means that anything that can induce an OCB in the employees of a firm will lead to lower turnover intentions of those employees including peripheral CSR. Therefore, retaining employees for longer periods will demand firms to work on OCB at their workplace (Yan et al., 2021). An organization comprises people and their retention is foremost important for successful organizations, as employee turnover is a very costly phenomenon for the organizations (Aguilera et al., 2007). Peripheral CSR and CSR for the community lead to important benefits for the organization (Aman et al., 2021); however, embedding CSR at the activity level holds more promise as far as the employees of the firm are concerned and eventually can contribute toward addressing sustainability challenges.

## Conclusion

The research analyses 297 responses from employees of various firms renowned for their CSR initiatives in Pakistan. Using the PLS-SEM, we observe strong support for the direct and mediating effects of the embedded CSR activities on the employee turnover intention as compared to the peripheral CRS activities. These results provide evidence for firms that are interested in retaining their employees and that indicate the role of embedded CSR, peripheral CSR, and OCB in doing so. Embedding CSR in the daily activities of the firm has more value as compared to just performing CSR activities for the sake of marketing and positioning as a “good” firm.

The present research carries certain limitations that will lead to new avenues for the researchers to explore further. We collected only cross-sectional data for the study; however, the generalization would be better off with multiple observations across various times. Second, we believe that these dimensions should also be linked to other important variables such as managerial support and organizational culture to investigate the big picture across the two CSR dimensions.

Finally, since all of our individuals are from collectivist cultures, our findings might be different in another cultural setting. In a collectivist culture, everyone is expected to look out for one another and the “We” takes precedence over the “I” (Hofstede, 2001). There is a dearth of cross-cultural micro-CSR research conducted as per our findings (Vlachos et al., 2014), and these reveal that reaction in highly individualistic cultures is different, as compared to in collectivist culture, to tradeoffs between the self- and other-directed CSR activities by the stakeholders.

When the other-directed CSR targets employees whose self-interest does not coincide with the in-group’s interest, it is a good possibility that other-orientation in highly individualistic environments will have a bigger part to play.

## Data Availability Statement

The raw data supporting the conclusions of this article will be made available by the authors, without undue reservation.

## Author Contributions

YL: overall supervision, funding, and conceptualization. KI: conceptualization and writing. MH: analysis, methodology, and editing. AK: literature review and introduction. ZA: conceptualization, discussion, and results. All authors contributed to the article and approved the submitted version.

## Conflict of Interest

The authors declare that the research was conducted in the absence of any commercial or financial relationships that could be construed as a potential conflict of interest.

## Publisher’s Note

All claims expressed in this article are solely those of the authors and do not necessarily represent those of their affiliated organizations, or those of the publisher, the editors and the reviewers. Any product that may be evaluated in this article, or claim that may be made by its manufacturer, is not guaranteed or endorsed by the publisher.

## References

[B1] Abbas J. Mahmood S. Ali H. Ali Raza M. Ali G. Aman J. (2019a). The effects of corporate social responsibility practices and environmental factors through a moderating role of social media marketing on sustainable performance of business firms. *Sustainability* 11:3434. 10.3390/su11123434

[B2] Abbas J. Raza S. Nurunnabi M. Minai M. S. Bano S. (2019b). The impact of entrepreneurial business networks on firms’ performance through a mediating role of dynamic capabilities. *Sustainability* 11:3006. 10.3390/su11113006

[B3] Abbas J. Zhang Q. Hussain I. Akram S. Afaq A. Shad M. A. (2020). Sustainable innovation in small medium enterprises: the impact of knowledge management on organizational innovation through a mediation analysis by using SEM approach. *Sustainability* 12:2407. 10.3390/su12062407

[B4] Afzal F. Yunfei S. Junaid D. Hanif M. S. (2020). Cost-risk contingency framework for managing cost overrun in metropolitan projects: using fuzzy-AHP and simulation. *Int. J. Manag. Proj. Bus*. 13 1121–1139. 10.1108/IJMPB-07-2019-0175

[B5] Aguilera R. V. Rupp D. E. Williams C. A. Ganapathi J. (2007). Putting the S back in corporate social responsibility: a multilevel theory of social change in organizations. *Acad. Manage. Rev.* 32 836–863. 10.5465/amr.2007.25275678

[B6] Aguinis H. (2011). “Organizational responsibility: doing good and doing well,” in *APA Handbook of Industrial and Organizational Psychology, Maintaining, Expanding, and Contracting the Organization*, Vol. 3, ed. Zedeck S. (Washington, DC: American Psychological Association), 855–879.

[B7] Aguinis H. Glavas A. (2012). What we know and don’t know about corporate social responsibility: a review and research agenda. *J. Manage.* 38 932–968. 10.1177/0149206311436079

[B8] Aman J. Abbas J. Shi G. Ain N. U. Gu L. (2021). Community wellbeing under China-Pakistan economic corridor: role of social, economic, cultural, and educational factors in improving residents’ quality of life. *Front. Psychol*. 12:816592. 10.3389/fpsyg.2021.816592 35422725 PMC9004670

[B9] Ang R. Shao Z. Liu C. Yang C. Zheng Q. (2022). The relationship between CSR and financial performance and the moderating effect of ownership structure: evidence from Chinese heavily polluting listed enterprises. *Sustain. Prod. Consum.* 30 117–129. 10.1016/j.spc.2021.11.030

[B10] Asad A. Abbas J. Irfan M. Raza H. M. A. (2017). The impact of HPWS in organizational performance: a mediating role of servant leadership. *J. Manage. Sci*. 11 25–48.

[B11] Asada A. Basheerb M. F. Irfanc M. Jiangd J. Tahir R. (2020). Open-Innovation and knowledge management in Small and Medium-Sized Enterprises (SMEs): the role of external knowledge and internal innovation. *Rev. Argent. Clin. Psicol.* 29 80–90.

[B12] Azizi M. R. Atlasi R. Ziapour A. Abbas J. Naemi R. (2021). Innovative human resource management strategies during the COVID-19 pandemic: a systematic narrative review approach. *Heliyon* 7:e07233. 10.1016/j.heliyon.2021.e07233 34124399 PMC8183111

[B13] Badar M. S. Irfan M. (2018). Shopping mall services and customer purchase intention along with demographics. *J. Mark. Focus. Manage.* 1–17.

[B14] Barnett M. L. (2007). Stakeholder influence capacity and the variability of financial returns to corporate social responsibility. *Acad. Manage. Rev.* 32 794–816. 10.5465/amr.2007.25275520

[B15] Bothma C. F. C. Roodt G. (2013). The validation of the turnover intention scale. *SA J. Hum. Resour. Manage.* 11 1–12. 10.4102/sajhrm.v11i1.507

[B16] Brammer S. Millington A. Rayton B. (2007). The contribution of corporate social responsibility to organizational commitment. *Int. J. Hum. Resour. Manage.* 18 1701–1719. 10.1080/09585190701570866

[B17] Bridoux F. Stofberg N. Den Hartog D. (2016). Stakeholders’ responses to csr tradeoffs: when other-orientation and trust trump material self-interest. *Front. Psychol.* 6:1992. 10.3389/fpsyg.2015.01992 26834657 PMC4712297

[B18] Burbano V. C. (2016). Social responsibility messages and worker wage requirements: field experimental evidence from online labor marketplaces. *Organ. Sci.* 27 1010–1028. 10.1287/orsc.2016.1066 19642375

[B19] Carnahan S. Kryscynski D. Olson D. (2017). When does corporate social responsibility reduce employee turnover? Evidence from attorneys before and after 9/11. *Acad. Manage. J.* 60 1932–1962. 10.5465/amj.2015.0032

[B20] Carroll A. B. (1979). A three-dimensional conceptual model of corporate performance. *Acad. Manage. Rev.* 4 497–505. 10.1002/ar.23726 29150982

[B21] Carroll A. B. Shabana K. M. (2010). The business case for corporate social responsibility: a review of concepts, research and practice. *Int. J. Manage. Rev.* 12 85–105. 10.1111/j.1468-2370.2009.00275.x

[B22] Chen X.-P. Hui C. Sego D. J. (1998). The role of organizational citizenship behavior in turnover: conceptualization and preliminary tests of key hypotheses. *J. Appl. Psychol.* 83 922–931. 10.1037/0021-9010.83.6.922

[B23] Chitsaz E. Liang D. Khoshsoroor S. (2017). The impact of resource configuration on Iranian technology venture performance. *Technol. Forecast. Soc. Change* 122 186–195. 10.1016/j.techfore.2016.03.009

[B24] Clarkson M. E. (1995). A stakeholder framework for analyzing and evaluating corporate social performance. *Acad. Manage. Rev.* 20 92–117. 10.2307/258888

[B25] De Gilder D. Schuyt T. N. M. Breedijk M. (2005). Effects of an employee volunteering program on the work force: the ABN-AMRO case. *J. Bus. Ethics* 61 143–152. 10.1007/s10551-005-7101-x

[B26] De Roeck K. Delobbe N. (2012). Do environmental CSR initiatives serve organizations’ legitimacy in the oil industry? Exploring employees’ reactions through organizational identification theory. *J. Bus. Ethics* 110 397–412. 10.1007/s10551-012-1489-x

[B27] Desiderio E. García-Herrero L. Hall D. Segrè A. Vittuari M. (2021). Social sustainability tools and indicators for the food supply chain: a systematic literature review. *Sustain. Prod. Consum*. 30 527–540. 10.1016/j.spc.2021.12.015

[B28] El Akremi A. Gond J.-P. Swaen V. De Roeck K. Igalens J. (2018). How do employees perceive corporate responsibility? Development and validation of a multidimensional corporate stakeholder responsibility scale. *J. Manage.* 44 619–657. 10.1177/0149206315569311

[B29] Fishbein M. Ajzen I. (1977). Belief, attitude, intention, and behavior: an introduction to theory and research. *Philos. Rhetor*. 10 130–132. 10.1007/s00267-013-0054-4 23609308

[B30] Flammer C. Luo J. (2017). Corporate social responsibility as an employee governance tool: evidence from a quasi−experiment. *Strateg. Manage. J.* 38 163–183. 10.1002/smj.2492

[B31] Fornell C. Larcker D. (1981). Evaluating structural equation models with unobservable variables and measurement error. *J. Mark. Res.* 18 39–50. 10.2307/3151312

[B32] Freeman R. (1984). *Strategic Management: A Stakeholder Approach.* Boston, MA:Pitman.

[B33] Freeman R. E. (2010). *Strategic Management: A Stakeholder Approach.* Cambridge: Cambridge university press. 10.1017/CBO9781139192675

[B34] Freeman R. E. Phillips R. Sisodia R. (2020). Tensions in stakeholder theory. *Bus. Soc.* 59 213–231. 10.1177/0007650318773750

[B35] Frynas J. G. Yamahaki C. (2016). Corporate social responsibility: review and roadmap of theoretical perspectives. *Bus. Ethics A Eur. Rev.* 25 258–285. 10.1111/beer.12115

[B36] Fu Q. Abbas J. Sultan S. (2022). Reset the industry redux through corporate social responsibility: the COVID-19 tourism impact on hospitality firms through business model innovation. *Front. Psychol*.

[B37] Ge T. Abbas J. Raza Ullah A. A. Sadiq I. Zhang R. (2022). Women’s entrepreneurial contribution to family income: innovative technologies promote females’ entrepreneurship amid COVID-19 crisis. *Front. Psychol*. 13:828040. 10.3389/fpsyg.2022.828040 35422737 PMC9004668

[B38] Greening D. W. Turban D. B. (2000). Corporate social performance as a competitive advantage in attracting a quality workforce. *Bus. Soc.* 39 254–280. 10.1177/000765030003900302

[B39] Hair J. F. (2009). *Multivariate Data Analysis*, 7th Edn. Upper Saddle River, NJ: Prentice Hall.

[B40] Hanif M. S. Hanif M. I. Shao Y. (2018). Contemplating the antecedents of a sustainable work life in an emerging economy: lessons from early retirees in the ICT sector of Pakistan. *Sustainability* 10:4734. 10.3390/su10124734

[B41] Hanif M. S. Wang M. Mumtaz M. U. Ahmed Z. Zaki W. (2021). What attracts me or prevents me from mobile shopping? An adapted UTAUT2 model empirical research on behavioral intentions of aspirant young consumers in Pakistan. *Asia Pac. J. Mark. Logist.* 34 1031–1059. 10.1108/APJML-09-2020-0659

[B42] Hansen S. D. Dunford B. B. Boss A. D. Boss R. W. Angermeier I. (2011). Corporate social responsibility and the benefits of employee trust: a cross-disciplinary perspective. *J. Bus. Ethics* 102 29–45. 10.1007/s10551-011-0903-0

[B43] He J. Zhang H. Morrison A. M. (2019). The impacts of corporate social responsibility on organization citizenship behavior and task performance in hospitality: a sequential mediation model. *Int. J. Contemp. Hosp. Manage.* 31 2582–2598. 10.1108/IJCHM-05-2018-0378

[B44] Hofstede G. (2001). *Culture’s Consequences: Comparing Values, Behaviors, Institutions and Organizations Across Nations*, 2nd Edn. Thousand Oaks, CA: Sage Publications.

[B45] Hollingworth D. Valentine S. (2014). Corporate social responsibility, continuous process improvement orientation, organizational commitment and turnover intentions. *Int. J. Qual. Reliab. Manage*. 31 629–651. 10.1108/IJQRM-09-2012-0131

[B46] Jones D. A. (2010). Does serving the community also serve the company? Using organizational identification and social exchange theories to understand employee responses to a volunteerism programme. *J. Occup. Organ. Psychol.* 83 857–878. 10.1348/096317909X477495

[B47] Katz D. (1964). The motivational basis of organizational behavior. *Behav. Sci.* 9 131–146. 10.1002/bs.3830090206 5888769

[B48] Khurshid A. Ahmed A. (2020). Turning suppliers into sustainable agents of the firm. *Paradigms*. SI, 47–52.

[B49] Kock N. (2015). Common method bias in PLS-SEM: a full collinearity assessment approach. *Int. J. eCollab.* 11 1–10. 10.4018/ijec.2015100101

[B50] Kock N. Hadaya P. (2018). Minimum sample size estimation in PLS−SEM: the inverse square root and gamma−exponential methods. *Inf. Syst. J.* 28 227–261. 10.1111/isj.12131

[B51] Kolling C. Ribeiro J. L. D. de Medeiros J. F. (2022). Performance of the cosmetics industry from the perspective of Corporate Social Responsibility and Design for Sustainability. *Sustain. Prod. Consum.* 30 171–185. 10.1016/j.spc.2021.12.002

[B52] Kong Y. Javed F. Sultan J. Hanif M. S. Khan N. (2022). EMA implementation and corporate environmental firm performance: a comparison of institutional pressures and environmental uncertainty. *Sustainability* 14:5662. 10.3390/su14095662.

[B53] Kristof−Brown A. L. Zimmerman R. D. Johnson E. C. (2005). Consequences OF INDIVIDUALS’FIT at work: a meta−analysis OF person–job, person–organization, person–group, and person–supervisor fit. *Pers. Psychol.* 58 281–342. 10.1111/j.1744-6570.2005.00672.x

[B54] Kumar M. M. Shah S. A. (2015). Psychometric properties of Podsakoff’s organizational citizenship behaviour scale in the Asian context. *Int. J. Indian Psychol.* 3 51–60. 10.25215/0301.152

[B55] Lacity M. C. Iyer V. V. Rudramuniyaiah P. S. (2009). “Turnover intentions of Indian IS professionals,” in *Information Systems Outsourcing*, eds Hirschheim R. Heinzl A. Dibbern J. (Berlin: Springer), 393–421. 10.1371/journal.pone.0229954

[B56] Li Y. Xie W. (2021). Linking change-oriented organizational citizenship behavior to turnover intention: effects of servant leadership and career commitment. *Public Pers. Manage.* 51 3–23.

[B57] Liu Q. Qu X. Wang D. Abbas J. Mubeen R. (2021). Product market competition and firm performance: business survival through innovation and entrepreneurial orientation amid COVID-19 financial crisis. *Front. Psychol*. 12:790923. 10.3389/fpsyg.2021.790923 35411208 PMC8993680

[B58] Lowery C. M. Krilowicz T. J. (1996). Effects of organizational citizenship behaviours: evidence from production supervisors. *Int. J. Sel. Assess.* 4 18–23. 10.1037/0021-9010.86.4.789 11519662

[B59] Manoppo V. P. (2020). Transformational leadership as a factor that decreases turnover intention: a mediation of work stress and organizational citizenship behavior. *TQM J.* 32 1395–1412.

[B60] Mitchell R. K. Agle B. R. Wood D. J. (1997). Toward a theory of stakeholder identification and salience: defining the principle of who and what really counts. *Acad. Manage. Rev.* 22 853–886.

[B61] Moorman R. H. (1991). Relationship between organizational justice and organizational citizenship behaviors: Do fairness perceptions influence employee citizenship? *J. Appl. Psychol.* 76 845.

[B62] Mubeen R. Han D. Abbas J. Álvarez-Otero S. Sial M. S. (2021). The relationship between CEO duality and business firms’ performance: the moderating role of firm size and corporate social responsibility. *Front. Psychol.* 12:669715. 10.3389/fpsyg.2021.669715 35035363 PMC8757377

[B63] Muzaffar A. Khurshid A. Malik M. N. Azhar A. (2019). Sustainable development across the supply chain: the missing link of socio−environmental effect. *Sustain. Dev.* 27 976–981.

[B64] Oates G. Kloot L. (2014). Corporatized public land development bodies in Australia: Who are the stakeholders and why are they important? *Int. J. Public Adm.* 37 163–173.

[B65] Organ D. W. (1988). *Organizational Citizenship Behavior: The Good Soldier Syndrome*. Lexington, MA: Lexington books.

[B66] Organ D. W. Konovsky M. (1989). Cognitive versus affective determinants of organizational citizenship behavior. *J. Appl. Psychol.* 74 157–164.

[B67] Osgood C. E. (1964). Semantic differential technique in the comparative study of cultures. *Am. Anthropol.* 66 171–200.

[B68] Peloza J. Shang J. (2011). How can corporate social responsibility activities create value for stakeholders? A systematic review. *J. Acad. Mark. Sci.* 39 117–135.

[B69] Podsakoff P. M. Ahearne M. MacKenzie S. B. (1997). Organizational citizenship behavior and the quantity and quality of work group performance. *J. Appl. Psychol.* 82 262–270. 10.1037/0021-9010.82.2.262 9109284

[B70] Podsakoff P. M. MacKenzie S. B. Lee J.-Y. Podsakoff N. P. (2003). Common method biases in behavioral research: a critical review of the literature and recommended remedies. *J. Appl. Psychol.* 88 879–903. 10.1037/0021-9010.88.5.879 14516251

[B71] Post J. E. Preston L. E. Sauter-Sachs S. (2002). *Redefining the Corporation: Stakeholder Management and Organizational Wealth.* Stanford, CA: Stanford University Press.

[B72] Qing C. Jin S. (2022). How does corporate social responsibility affect sustainability of social enterprises in Korea? *Front. Psychol.* 13:859170. 10.3389/fpsyg.2022.859170 35310265 PMC8924114

[B73] Rasoolimanesh S. M. Jaafar M. Kock N. Ahmad A. G. (2017). The effects of community factors on residents’ perceptions toward World Heritage Site inscription and sustainable tourism development. *J. Sustain. Tour.* 25 198–216.

[B74] Reynolds S. J. Schultz F. C. Hekman D. R. (2006). Stakeholder theory and managerial decision-making: constraints and implications of balancing stakeholder interests. *J. Bus. Ethics* 64 285–301.

[B75] Rupp D. E. Ganapathi J. Aguilera R. V. Williams C. A. (2006). Employee reactions to corporate social responsibility: an organizational justice framework. *J. Organ. Behav.* 27 537–543.

[B76] Rupp D. E. Shao R. Thornton M. A. Skarlicki D. P. (2013). Applicants’ and employees’ reactions to corporate social responsibility: the moderating effects of first−party justice perceptions and moral identity. *Pers. Psychol.* 66 895–933.

[B77] Rupp D. E. Williams C. A. Aguilera R. V. (2011). “Increasing corporate social responsibility through stakeholder value internalization (and the catalyzing effect of new governance): an application of organizational justice, self-determination, and social influence theories,” in *Managerial Ethics: Managing the Psychology Morality*, ed. Schminke M. (New York, NY: Routledge), 69–88.

[B78] Skarlicki D. P. Latham G. P. (1995). Organizational citizenship behaviour and performance in a university setting. *Can. J. Adm. Sci.* 12 175–181.

[B79] Smith C. Organ D. W. Near J. P. (1983). Organizational citizenship behavior: Its nature and antecedents. *J. Appl. Psychol.* 68 653–663. 10.1037/0021-9010.68.4.653

[B80] Soper D. S. (2020). *A-Priori Sample Size Calculator for Structural Equation Models [Software].* Available online at: https://www.danielsoper.com/statcalc (accessed January 03, 2022).

[B81] Tett R. P. Meyer J. P. (1993). Job satisfaction, organizational commitment, turnover intention, and turnover: path analyses based on meta−analytic findings. *Pers. Psychol.* 46 259–293. 10.1111/j.1744-6570.1993.tb00874.x

[B82] Turban D. B. Greening D. W. (1997). Corporate social performance and organizational attractiveness to prospective employees. *Acad. Manage. J.* 40 658–672. 10.5465/257057

[B83] Turker D. (2009). Measuring corporate social responsibility: a scale development study. *J. Bus. Ethics* 85 411–427. 10.1007/s10551-008-9780-6

[B84] Vlachos P. A. Panagopoulos N. G. Theotokis A. Singh R. Singh R. K. (2014). When do corporate social responsibility initiatives impact on customer-facing employees? Evidence from India and the Netherlands. *Int. J. Hum. Resour. Manage.* 25 3086–3112. 10.1080/09585192.2014.934884

[B85] Wang W. Fu Y. Qiu H. Moore J. H. Wang Z. (2017). Corporate social responsibility and employee outcomes: a moderated mediation model of organizational identification and moral identity. *Front. Psychol.* 8:1906. 10.3389/fpsyg.2017.01906 29163287 PMC5671997

[B86] Yan A. Tang L. Hao Y. (2021). Can corporate social responsibility promote employees’ taking charge? The mediating role of thriving at work and the moderating role of task significance. *Front. Psychol*. 11:613676. 10.3389/fpsyg.2020.613676 33584449 PMC7875869

[B87] Zhang M. Di Fan D. Zhu C. J. (2014). High-performance work systems, corporate social performance and employee outcomes: exploring the missing links. *J. Bus. Ethics* 120 423–435. 10.1007/s10551-013-1672-8

[B88] Zhang Q. Irfan M. Khattak M. A. O. Abbas J. Zhu X. Shah M. S. (2012). Critical success factors for successful lean six sigma implementation in Pakistan. *Interdiscip. J. Contemp. Res. Bus.* 4 117–124. 10.1371/journal.pone.0225669 31774862 PMC6881029

[B89] Zhou Y. Draghici A. Abbas J. Mubeen R. Boatca M. E. Salam M. A. (2021). Social media efficacy in crisis management: effectiveness of non-pharmaceutical interventions to manage COVID-19 challenges. *Front. Psychiatry* 12:626134. 10.3389/fpsyt.2021.626134 35197870 PMC8859332

